# Extracellular Matrix Remodeling of Adipose Tissue in Obesity and Metabolic Diseases

**DOI:** 10.3390/ijms20194888

**Published:** 2019-10-02

**Authors:** Francisco Javier Ruiz-Ojeda, Andrea Méndez-Gutiérrez, Concepción María Aguilera, Julio Plaza-Díaz

**Affiliations:** 1 Department of Biochemistry and Molecular Biology II, School of Pharmacy, University of Granada, 18071 Granada, Spaincaguiler@ugr.es (C.M.A.); 2 Instituto de Investigación Biosanitaria IBS.GRANADA, Complejo Hospitalario Universitario de Granada, 18014 Granada, Spain; 3 RG Adipocytes and metabolism, Institute for Diabetes and Obesity, Helmholtz Diabetes Center at Helmholtz Center Munich, 85764 Neuherberg, Munich, Germany; 4 Institute of Nutrition and Food Technology "José Mataix", Center of Biomedical Research, University of Granada, Avda. del Conocimiento s/n., 18016 Armilla, Granada, Spain; 5 CIBEROBN (CIBER Physiopathology of Obesity and Nutrition), Instituto de Salud Carlos III, 28029 Madrid, Spain

**Keywords:** obesity, adipose tissue, extracellular matrix, insulin resistance

## Abstract

The extracellular matrix (ECM) is a network of different proteins and proteoglycans that controls differentiation, migration, repair, survival, and development, and it seems that its remodeling is required for healthy adipose tissue expansion. Obesity drives an excessive lipid accumulation in adipocytes, which provokes immune cells infiltration, fibrosis (an excess of deposition of ECM components such as collagens, elastin, and fibronectin) and inflammation, considered a consequence of local hypoxia, and ultimately insulin resistance. To understand the mechanism of this process is a challenge to treat the metabolic diseases. This review is focused at identifying the putative role of ECM in adipose tissue, describing its structure and components, its main tissue receptors, and how it is affected in obesity, and subsequently the importance of an appropriate ECM remodeling in adipose tissue expansion to prevent metabolic diseases.

## 1. Introduction

The increase in overweight and obesity prevalence is a result of lifestyle changes that are due to the social and demographic transition that started some decades ago and it has been dramatically augmented in the worldwide [1,2,3]. It was already recognized that there are individuals who despite having normal weight, they have an increased metabolic and cardiovascular risk due to they are hyperinsulinemic, insulin resistant, hypertriglyceridemic and predisposed to subsequent development of type 2 diabetes (T2D) [4]. Additionally, being normal weight metabolically unhealthy in childhood predicts lower insulin sensitivity as youth enter puberty [5].

The extracellular matrix (ECM) is a complex structure composed by different proteins, proteoglycans and polysaccharides, which provides a scaffold for cells modulating biological processes such as cell adhesion, migration, repair, survival, and development. The role of ECM in cell adhesion and signaling into the cells is carried out by integrins, which transduce signals through the plasma membrane to activate intracellular signaling [6,7,8]. As obesity is characterized by massive adipose tissue expansion, ECM remodeling and reorganization are requisites to providing enough space for the enlargement of adipocytes (hypertrophy), and to form new ones through adipogenesis from the precursor cells (hyperplasia) [9]. In adipose tissue, ECM is composed mainly by collagens (I, II, III, and IV), fibronectin, and a small amount of laminin [10,11]. However, several components, such as A disintegrin and metalloproteinase domain-containing protein (ADAMs), osteopontin (OPN), hyaluronan (HA), thrombospondins (THBS1), matrix metalloproteases (MMPs), and tissue inhibitor of metalloproteinases (TIMPs), play an important role in the ECM remodeling and adipose tissue function [12,13]. Besides, this process allows the formation of new blood vessels, which is crucial for the healthy adipose tissue expansion because the failure of this results in necrosis of adipocytes, and hypoxia, which triggers chronic, low-grade inflammation and fibrosis, which is a major player in adipose tissue dysfunction, and lastly insulin resistance (IR) [9,14,15]. Indeed, the ECM and integrins are important regulators of insulin action and it may be a novel therapeutic target to treat the underlying IR associated with T2D [6]. The aim of this review is to update the importance of ECM remodeling in adipose tissue to prevent adipocyte dysfunction, and then the fibrosis, inflammation, IR related to obesity and metabolic diseases.

## 2. Structure of Extracellular Matrix in the Adipose Tissue and Obesity

### 2.1. Integrins and Other Receptors

Integrins are the major tissue receptors for cell adhesion to ECM proteins, and also play important roles in certain cell–cell adhesions. Since they were recognized by Hynes in 1987 [16], they have intensively studied as adhesion receptors, and they transduce signals through the plasma membrane to activate intracellular signaling. Integrins are heterodimeric transmembrane receptors composed by α- and β-subunits, and so far they can assemble into 24 distinct integrins, with different ligand-binding specificities and signaling properties [8]. Integrins are composed of a large ectodomain, which mediates ligand binding; a transmembrane domain; and a short cytoplasmic tail, which indirectly associates with the actomyosin cytoskeleton. For their activation, it is required a shift from bent-closed/extended-closed conformation to the extended-open conformation. To do this, there are intracellular adaptor proteins, like talins and kindlins, which are indispensably implicated in the integrin activation [17,18]. However, integrins themselves lack kinase activity, and the downstream signaling is through focal adhesion kinase (FAK) and integrin-linked kinase (ILK). FAK is a tyrosine kinase with the properties of intracellular signaling, stabilization of cytoskeleton, and focal adhesion turnover, and it is regulated by the epidermal growth factor receptor (EGFR), fibroblast growth factor receptor (FGFR), and the insulin receptor (IRc). In adipose tissue, FAK signaling controls insulin sensitivity through regulation of adipocyte survival in adipose tissue [19], and ILK interacts with β1, β2, and β3-integrin cytoplasmic domains and numerous cytoskeleton-associated proteins. Collagen, fibronectin, laminin, Arg–Gly–Asp peptide (RGD), and leucocytes all bind integrin receptors, and collagen and laminin share a common integrin β1 subunit, whereas leucocytes bind to integrin β2 subunit [6].

**Figure 1:**
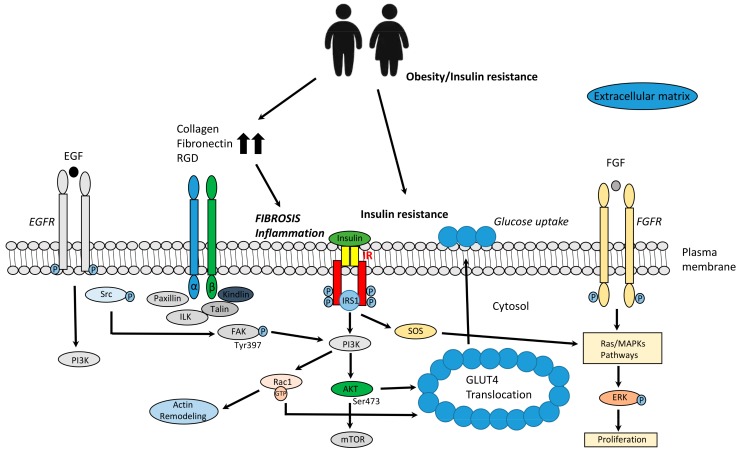
ECM remodeling is linked to obesity and IR in adipose tissue. Abbreviations: AKT: protein kinase B; EGF: epidermal growth factor; EGFR: epidermal growth factor receptor; ERK: extracellular-signal-regulated kinase; FGF: fibroblast growth factor; FGFR: fibroblast growth factor receptor; GLUT4: glucose transporter type 4; ILK: integrin-linked kinase; IR: insulin receptor; IRS1: Insulin receptor substrate 1; MAPK: mitogen-activated protein kinases; mTOR: mammalian target of Rapamycin; PI3K: phosphatidylinositol 3-kinase; RGD: Arg–Gly–Asp peptide; SOS: Son of Sevenless; Src: Proto-oncogene tyrosine-protein kinase.

Some studies suggest cross-talk between ECM and insulin signaling; indeed, striated muscle-specific integrin β1-deficient mice show IR by impairment of insulin-stimulated skeletal muscle glucose uptake and glycogen synthesis resulted from a decrease in AKT Ser-473 phosphorylation [20]. Moreover, diet-induced muscle IR is associated with ECM and interaction with integrin α2β1 in mice, therefore this data support an important cross-talk between integrin receptor function and insulin action in skeletal muscle [21]. Although the function of integrins in adipose tissue is still unknown, nonetheless, there are some studies indicating an important role. Actually, adipose-specific loss of kindlin-2, which promotes integrin activation, provokes lipodystrophy and metabolic disturbance [22]. In transgenic and T2D animal models, integrin β1 is displayed as modulator of GLUT4, the most important insulin-dependent glucose transporter in adipose tissue. In addition, the ILK has been suggested to modulate capillarization of the muscle from diet-induced insulin resistant mice [23]. In mice with general depletion of ILK, in adulthood, the integrin signaling molecule exhibits hyperglycemia and hyperinsulinemia with a downregulation in GLUT4 expression, decreasing the insulin sensitivity and AKT phosphorylation at Ser473, suggesting that ILK may be a molecular target and a prognostic biomarker of IR [24] (Figure 1). It has been also identify the mechanical stress as a novel mechanism directly regulating Drosophila insulin sensitivity and resistance. The mechanical stress caused by agitation of tissue ex vivo or body movement in vivo is required for activation of insulin signaling in the Drosophila larval fat body and this movement induces the membrane localization of the IRc and several IRc substrates. Sensing of mechanical stimuli is mediated in part by integrins, whose activation is necessary and sufficient for mechanical stress-dependent insulin signaling, suggesting that integrin signaling and plays a crucial role in the membrane localization of IRc to regulate insulin sensitivity [25]. On the other hand, there are integrins like β2, which are implicated in the immune system due to they are key in trafficking and function of leukocytes. These integrins β2 are regulated by kindlin-3 and talin as cytoplasmic intracellular domains and those integrins increase neutrophil production and infiltration into muscle, which aggravate the IR state. Therefore, integrins β2 modulates glucose homeostasis under high-fat diet (HFD) feeding, predominantly through actions on skeletal muscle and adipose tissue [26].

The CD44 is a cell surface transmembrane glycoprotein ubiquitously expressed and it binds to the ECM, mainly HA and OPN. CD44 regulates different cell functions like cell–cell and cell–matrix interactions [27], and it has been described three types of molecular action: first, it can bind different ligands, such as HA, which drives cell behavior regardless the interactions with receptor tyrosine kinase or actin cytoskeleton. Second, CD44 has coreceptor functions that mediate the signaling of receptor tyrosine kinases; and, third, CD44 provides a link between the plasma membrane and the actin cytoskeleton [28]. Regarding obesity and metabolic diseases, CD44 plays an important role in development of adipose tissue inflammation and IR. CD44 deficiency ameliorates IR and adipose tissue inflammation in a diabetic mouse model, and the treatment with an anti-CD44 antibody decreases blood glucose levels and macrophages infiltration under high-fat diet (HFD) [29]. Indeed, a recent study showed that treatment with HA nanoparticles in diet-induced obesity mice suppressed adipose tissue inflammation as indicated by reduced macrophage content, the production of proinflammatory cytokines and NLRP3 inflammasome activity in epididymal white adipose tissue (WAT), leading to improved insulin sensitivity and normalized blood glucose levels [30]. In humans, CD44 is highly expressed in inflammatory cells in obese adipose tissue and serum levels are positively correlated with IR and glycemic control. Moreover, OPN and CD44 gene expression is increased in human obese adipose tissue, representing a potential therapeutic target for treating IR [31]. On the other hand, a genome-wide DNA methylation analysis showed an epigenetic regulation involved in the dysregulation of visceral adipose tissue in humans. The authors differentiated between insulin-resistant from insulin-sensitive obese subjects, and CD44 was identified as a novel IR-related gene that could predispose patients to IR and future T2D in morbid obesity [32].

### 2.2. Collagens

Collagen is the main ECM component and contributes considerably to the non-cell mass of the adipose tissue. Collagen is primarily produced by the adipocytes, although the preadipocytes, endothelial cells and the stem cells can also produce it. Mature adipocytes store energy as triglycerides, and this drives a strong mechanical stress, which is transferred from the outside to the inside of the cell and can be decreased by the strong external skeleton. Furthermore, collagens contribute to cell adhesion, migration, differentiation, morphogenesis, and wound healing in the adipose tissue. Between the collagens, collagen IV is a major component in each adipocyte as basement membrane, and this is necessary for adipocyte survival [13,33]. Collagen I is the most abundant component of ECM [34]. In obesity, accumulation of collagen causes fibrosis of adipose tissue increasing rigidity, reduces its expandability, and provokes IR [35]. It has been observed in the adipose tissue of obese mice under HFD, such as collagen IV, which is highly increased in obese humans [36], that collagens I, III, V, and VI are increased [6,37].

In mice, collagen VI seems to be more specific for adipocytes. It is strongly binds to collagen IV, which is important for adipocyte survival and both collagens are classified as non-fibrillar types and their interaction has been suggested to mediate anchoring of the basement membrane to cells [33]. Collagens I, III, V and VI are increased in adipose tissue from obese mice under HFD [6,37]. Collagen type VI, α3 (COL6A3) is a protein highly expressed in mice adipose tissue and collagen IV KO lead to an enhancement in metabolic syndrome. Nevertheless, COL6A3 is downregulated in adipose tissue from obese people, whereas diet- and surgery-induced weight loss increases COL6A3 expression in subcutaneous WAT which is regulated by leptin treatment decreasing its expression [38]. In this line, Sun et al. (2014) demonstrated that endotrophin is a cleaved fragment of the α-3 chain of collagen VI, which has been shown to be implicated in the collagen deposition in adipose tissues during HFD exposure triggering inflammation and IR [39].

Finally, it has been described other collagen types such as collagen XVIII which is ubiquitously expressed and structural complex basement membrane proteoglycan which support preadipocyte differentiation and the maintenance of this differentiation state of adipocytes. Thus, a specific lack of this collagen in mice leads to reduced adiposity, ectopic lipid accumulation in liver, and increase very-low-density lipoprotein-triglyceride levels. Collagen XVIII was identified as extracellular matrix-directed mechanism that may contribute to the control of the multistep adipogenic program [40].

### 2.3. Matrix Metalloproteinases (MMPs) and Tissue Inhibitors of Metalloproteinases (TIMPs)

The metzincin superfamily of zinc-dependent metalloproteinases comprises the MMP, ADAM, and ADAMTS (ADAM with a Thrombospondin type-1 motif) subfamilies [41]. MMP is a family of calcium-dependent and zinc-containing endopeptidases that are responsible for the degradation of ECM proteins [42,43]. MMPs play an essential role in regulating ECM remodeling in both normal physiology and diseases and are principally involved in wound healing, angiogenesis, and tumor cell metastasis [43,44]. MMPs’ actions include other biological processes such as adipose tissue expansion, liver fibrosis, and atherosclerosis [45]. Besides, nuclear MMPs can induce apoptosis in cardiac myocytes, endothelial cells [46], and renal tubular cells [47], and several of them are able to bind to DNA promoters, regulating the transcription of either heat shock family proteins or different growth factors [48].

MMPs family members can be categorized into soluble collagenases (MMP1, -8, and -13), gelatinases A and B (MMP2 and -9), stromelysin-1, 2 and 3 (MMP3, -10, and -11), matrilysin-1 and -2 (MMP7 and -26), membrane-type MMPs (MT-MMPs) (MMP14, -15, -16, -17, -24, and -25), and elastase (MMP12) [44]. Endothelial cells, pericytes and podocytes, fibroblasts, and myofibroblats, and macrophages secrete MMP-2 and -9 [49]. Those MMPs degrade collagen IV and participate in vasculature remodeling, angiogenesis, inflammation, and atherosclerotic plaque rupture [50]. MMP-3 and MMP-10 have similar substrate specificities, although MMP-3 has higher proteolytic effects as compared to MMP-10. Both MMPs degrade fibronectin, laminin, gelatins-I, III, IV and V, collagen fibers, and proteoglycans. Others like MMP-7 and -26 are able to hydrolize fibronectin, gelatins and also they break human plasminogen generating a fragment that is angiogenesis inhibitor [51].

The MMPs are inhibited by specific endogenous TIMPs, which comprise a family of four protease inhibitors: TIMP-1, -2, -3, and -4 [52]. Circulating levels of TIMP-1 and -2 are increased in patients with metabolic syndrome and T2D [13,53]. MMPs imbalance is associated with the pathophysiology of obesity and T2D in humans [54,55,56]. Plasma concentrations of MMP-2 and -9 are increased in people with obesity [56] and T2D [53,57], but little information is available on the ADAMTS group [41].

The expansion of adipose tissue is associated with adipogenesis and angiogenesis [58] and different studies have demonstrated that MMPs are involved in both processes. The adipose expression of MMP-9 positively correlates with the homeostasis model assessment index of insulin resistance (HOMA-IR) in obese humans [56]. In animal models, MMP-3, MMP-11, MMP-12, MMP-13 and MMP-14 levels are upregulated in abdominal WAT, whereas MMP-7, MMP-9, MMP-16, MMP-24 and TIMP-4 were downregulated [59]. On the other hand, MMP-2 and MMP-9 activity are reduced in WAT from IR animal model induced by a sucrose-rich diet, and no changes were reported in MMP plasma activity [60,61]. In fact, a recent study has speculated that resistance training could play a key role in the maintenance of WAT ECM by modulating MMP-2, vascular endothelial growth factor (VEGF)-A, and TIMP-2 activity [62]. It has also been observed an increased level in WAT of MMP-9 in patients with obesity related to cardiovascular risk [63]. Although the specific role of these proteins in the development of obesity is not fully defined, MMPs gene targeting experiments in mice have identified variable functions of each protein in WAT [59,60,61,64,65].

The local balance between activated MMPs and TIMPs controls the net result of MMPs activity in tissues. However, this balance can be altered in some pathological situations leading to an uncontrolled activation of those MMPs [61]. On this matter, MMP-11 has also observed to be increased in the WAT of obese insulin-resistant mice, which suggested that dysregulation of MMP-11 may be an early process in tissue dysfunction [66]. Analyses of visceral and subcutaneous WAT from obese mice and humans have also pointed that upregulation of MMP-12 could be implied in obesity and IR development [67]. A study has shown that the deletion of MMP-12 exacerbated the HFD-induced hypertrophy, but improved insulin sensitivity [68]. Because of the upregulation of MMPs in obesity, a decrease in elastin is observed in obese WAT [13].

In relation to TIMPs dysregulation in obese adipose tissue, it has been observed an increase of TIMP-1 and -2 in patients with metabolic syndrome and diabetes [53]. In fact, these two enzymes may be considered as markers of non-alcoholic fatty liver disease (NAFLD) [69,70,71]. On the one hand, the authors reported an increase TIMP-1 levels in the serum of patients with gestational diabetes mellitus [72] and patients with obesity and cardiovascular risk [63]. Nevertheless, the overexpression of TIMP-1 in pancreatic β-cells pointed protection against diabetes in mice [73], whereas deletion of this protein provoked an increase in food intake and obesity [74]. These serum protein levels were also elevated in obese prediabetic rats [75]. On the other hand, the genetic deletion of TIMP-2 in mice promotes HFD-induced obesity and diabetes [76] and exercise has been reported to exert a positive effect in TIMP-2 modulation, improving insulin sensitivity [77]. Other TIMPs, such as TIMP-3 and -4, possess a crucial role in insulin sensitivity dysfunction. Specifically, a TIMP-3 deletion in mice has pointed to cause hepatic steatosis and WAT inflammation [78] while an overexpression seemed to protect from them [79]. Relating to TIMP-4, recent studies have evidenced the pathogenic effect of TIMP-4 deregulation in IR in different rodent models [80,81].

The expression of ADAMTS1, 4, 5, and 8 proteins in murine adipose tissue was detected, and a marked upregulation of ADAMTS5 during development of obesity was observed [82,83]; also in rat adipose tissue during HFD feeding [84]. In addition, Koza et al. have showed a positive correlation between *ADAMTS5* expression in adipose tissue and interindividual fat mass differences in genetically identical C57BL/6J obese mice, and also some authors have revealed that ADAMTS5 promotes murine adipogenesis and WAT expansion [85,86].

Elastin is a protein that confers elasticity to many tissues and it is degraded by MMPs [87]. In detail, MMP-12 (macrophage elastase) is one of the major MMPs degrading elastin in mice [88]. Under HFD, CD11c adipose macrophages (M2) express immense levels of MMP-12 [68,89]; although the literature supports that elastin downregulation aggravates IR in obese WAT [90,91].

ECM remodeling is composed of a bulk of processes and proteins, and further research is required for a better understanding and possible therapies development. TIMPs may act as endogenous inhibitors of MMPs that are responsible for degrading excess ECM, it is unclear whether the beneficial effects of increased TIMP or ADAMTS activities are solely due to the suppressed activity of MMPs and increased ECM stability [92]. Additional research is required for a major understanding of the implication of MMPs, TIMP, ADAMTS and elastin in metabolic disorders.

### 2.4. Other Components: Osteopontin, Hyaluronan, and Thrombospondin

The expression of OPN, another relevant component of ECM [93], is highly increased in the WAT of HFD-induced mice as well as people with obesity [94]. This protein is mostly expressed in WAT macrophages [95], and its deletion in mice has been demonstrated to prevent WAT inflammation and macrophage infiltration, and thus improve insulin sensitivity [96,97,98]. Recently, some studies have pointed out that plasma OPN is significantly elevated in T2D patients [99,100]. Relating to the possible key role of OPN in IR, it has also been proposed that baseline values of OPN may predict 3-year T2D remission in patients undergoing bariatric surgery [101]. In this study, authors observed baseline circulating levels of OPN significantly correlated with reductions of body weight, body mass index (BMI) and insulin sensitivity improvements [101].

Other components of WAT ECM are THBS1 and HA. The latter promotes monocyte adhesion and chemotaxis through the binding to CD44 [102,103]. HA is increased in obese mice comparing with their counterparts and the HA inhibitor treatment improved adipose inflammation and IR [104,105]. In humans, although some studies reinforced this theory [30,106,107], a recent study has signaled that HA decreases adipogenesis [108]. Thus, further studies are needed to elucidate the role of HA in IR. On the other hand, THBS1 is known to be highly increased in insulin-resistant obese mice and humans [109,110,111,112]. In mice, it has been shown that the treatment with recombinant THBS1 may suppress insulin signaling in the cultured muscle cell, which could represent crosstalk between the WAT and skeletal muscle in obesity [113]. On this matter, treatments against THBS1 may be a beneficial therapy against IR, even though further research is required.

## 3. Extracellular Matrix Remodeling of Adipose Tissue in Obesity and Insulin Resistance

### 3.1. Angiogenesis

Angiogenesis is the physiological process through which new blood vessels form from preexisting vessels, and it is essential for proper maintenance of normal tissue physiology and tissue remodeling and expansion [114,115]. It happens between the vascular (endothelial cells, pericytes and smooth muscle cells) and WAT components such as pre- or adipocytes, stromal vascular cells, fibroblasts, macrophages, and other proinflammatory cells [116,117]. These cells can secrete several pro- and antiangiogenic molecules to modulate angiogenesis through paracrine and autocrine mechanisms.

WAT is one of the most highly vascularized tissues in the body [118]. The blood vasculature is a closed tubular system that is arranged into tree-like structures composed of arteries, veins, and interconnecting capillaries [119]. Blood vessels provide oxygen, nutrients, hormones, cytokines, and growth factors to the tissue. It also supplies the infiltration of inflammatory cells and facilitates wasting products. Some studies have revealed that angiogenesis often precedes adipogenesis, being the expansion of WAT associated with active angiogenesis while inhibition of the latter prevents WAT enlargement, concluding the existence of a dynamic cross-talk between adipocytes and endothelial cells [120,121,122]. The current literature supports the essential role of impaired angiogenesis in WAT dysfunction. A recent work reported a lower gene expression in subcutaneous WAT of angiogenic markers, insulin sensitivity, and adipogenesis, whereas ECM remodeling markers were increased in obese and overweight subjects [123].

Although angiogenesis is a physiological process, it can be altered in some diseases such as obesity, diabetes, cancer, and cardiovascular diseases (CVD), playing a crucial role in these conditions [124]. In obesity, WAT expands and it consequently needs the formation of new vessels, which also promotes adipocyte differentiation [125]. However, hypertrophic growth of WAT is not often accompanied by a similar increased rate of angiogenesis, following dysfunction of the tissue [117,126]. The angiogenesis process is regulated by factors such as VEGF-A, and VEGF-B, fibroblast growth factor-2, angiopoietins 1-2 (Ang-1 and Ang-2), leptin, adiponectin, and plasminogen activator inhibitor-1, among others [127,128]. Especially, VEGF and, specifically, VEGF-A, through VEGF receptor-2 (VEGFR2), play a crucial role in the angiogenesis process [117,129,130].

On this matter, in spite of VEGF-A function has been quite studied in animal and human models; however, the results are ambiguous on the local and systemic levels of the protein during obesity due to its further metabolic effects. Several authors have reported an increase in VEGF serum concentration in overweight and obese subjects and animal models that also correlated with BMI [131,132,133]. In contrast, other studies have failed to reproduce these results [134]. On the contrary, other authors have observed a decrease in *Vegf* expression in WAT of obese mice [135,136] and obese humans [137]. These results and the fact that *Vegf* overexpression in mice was able to protect them from HFD induced inflammation and IR [138] are in accordance with a study that reported a higher VEGF levels in morbidly obese subjects with low IR than in obese subjects with high IR [56], supporting the idea of a close crosstalk between adipogenesis and angiogenesis [139]. A recent meta-analysis has also pointed out the strong association of an increase in VEGFs genes expression with metabolic syndrome, although evidence in obesity is confused [140]. Thus, a better understanding of VEGF-A actions on human metabolism and angiogenesis is needed.

On the other hand, it has been suggested that when WAT expansion occurs, hypertrophic adipocytes may become distant from the vasculature, generating hypoxic regions inside the tissue [141,142,143]. In this regard, hypoxia may represent a link between impaired adipogenesis and WAT inflammation due to the stabilization of the proinflammatory, factor hypoxia-inducible transcription factor-1 (HIF-1) and the consequent activation of proangiogenic factor such as VEGFs, Ang-1 and Ang-2, MMPs, leptin and plasminogen activators [117,141,144,145,146,147,148].

Even though hypoxia alone is not enough to stimulate angiogenesis, it has been suggested that it is one of the initiators of angiogenesis in animal models [141,149]. Nevertheless, the role of WAT hypoxia in human obesity is less compelling. Some studies support the theory observed previously [137,150,151], whereas others did not find the same results [152,153], and even report hyperoxic conditions during adipose tissue expansion [147]. Moreover, it has been observed that although obese insulin-resistant subjects present a reduced expression of angiogenic genes, along with decreased capillary density and blood flow in WAT [154], O_2_ partial pressure is unchanged [155,156,157]. The absence of hypoxia could be explained by the differences in methodology between studies as well as the reduced metabolic rate observed in the tissue [158]. However, it appears that hypoxia, despite not being the only angiogenic stimulus in WAT and the controversial implication in human obesity, plays a crucial role in angiogenesis and inflammation. A recent study has reported that the transmembrane glycoprotein CD248 affects several pathways related to hypoxia in adipocytes and modulates the vascularity of WAT, establishing a link between the lack of oxygen and angiogenesis. They also observed an increase in *CD248* expression in human white adipocytes that was positively associated with obesity and metabolic complications [159].

Finally, recent research denotes that modulation of angiogenic activity in WAT could result in benefits for obesity and metabolic disorders treatments [125]. Interestingly, novel subcutaneous implantation of the allograft adipose matrix with angiogenic and adipogenic factors has promoted adipogenesis in nude mouse and human dorsal wrist [160]. Thus, a better understanding of components of WAT and regulation of the microvasculature in human obesity would be of crucial importance to develop an effective treatment of obesity and associated disorders.

### 3.2. ECM Remodeling, Insulin Signaling, and Glucose Homeostasis

In obese WAT, both hypoxia and inflammation induce a pathological expansion of ECM with macrophages recruitment and increased protein expression, such as collagens [15,21,37,105,111,136,154,161,162,163,164,165]. This collagen accumulation hinders adipocyte expansion, which causes WAT to exceed its capacity to store fat and culminates in lipid deposition into other tissues, [161] such as the liver, skeletal muscle, pancreas, and heart [125]. It is known that an excess of tissue fat deposition promotes local inflammation and IR through the formation of different lipotoxic molecules [166]. Moreover, recent literature point out that ectopic lipid accumulation in the pancreas and kidneys may contribute to β-cell dysfunction, which could contribute to IR development [167,168]. Furthermore, an increase in visceral/intra-abdominal fat deposition is a marker of ectopic fat accumulation in various organs [169,170].

In particular, liver is one of the tissues with a greater predisposition for the lipid accumulation associated with dysfunctional WAT. NAFLD, whose prevalence is around 24%, is the most common chronic liver disease worldwide and obesity represents one of the most relevant risk factors [170]. The prevalence of this disease is of 80% in patients with obesity compared with 15% in healthy normal-weight individuals [171]. Although NAFLD increases with age in adults, it has been also reported in children and adolescents due to the high rates of obesity and T2D in these populations [172].

In this pathological condition, lipids are accumulated in the cytoplasm and give rise to lipid metabolites, leading to an imbalance between fatty acid oxidation, lipid disposal, and storage, inducing the synthesis of toxic lipid intermediates such as diacylglycerol and ceramides [173,174]. These compounds are associated with impaired insulin signaling and IR probably through the activation of hepatic protein kinase C [175,176]. Furthermore, adiponectin, an anti-inflammatory adipokine able to prevent lipid accumulation and with an insulin-sensitizing effect, is reduced in NAFLD, which aggravates IR in these patients [177,178]. Beyond insulin sensitivity state, an increase in hepatic lipid pool is also implied in the development of mitochondrial dysfunction, increased oxidative stress and the release of proinflammatory cytokines [179], what contributes to tissue inflammation.

Furthermore, it has been also suggested that early fat accumulation in the liver and hepatic IR precede skeletal muscle IR [180]. Therefore, even though it remains unclear if NAFLD is a cause or a consequence of IR [181,182], it is known that they are closely associated and hepatic lipid deposition is a risk factor for the development of CVD and T2D [183].

Additionally, skeletal muscle, one of the most important tissues in the body, represents 40% of total body mass and is an important regulator of glucose metabolism and lipid utilization [184]. As in the case of the liver, an excess in intramyocellular lipid (IMCL) accumulation is associated with the development of IR and T2D [185]. However, an accumulation of IMCLs has also been observed in highly trained insulin-sensitivity individuals, which also have a high oxidative capacity [186]. In this way, IR seems to be produced not only by skeletal muscle fat deposition, but also rather by the accumulation of the toxic lipid intermediates, such as diacylglycerol and ceramides.

Although fat deposition in the liver and skeletal muscle predominantly exerts a systemic IR effect, lipid accumulation in the epi-/pericardial areas, blood vessels, and myocardium itself seems to induce mostly local IR effects, further to contractile dysfunction, among others (reviewed in [187,188]). Besides, the epicardial fat depot is also suggested to release and secrete cytokines, adipokines and vasoactive factors to the adjacent myocardium and coronary arteries, thus contributing to CVD [142].

Although nonalcoholic fatty pancreas disease (NAFPD) has been less studied (reviewed in [189]), human studies have pointed that fat pancreatic accumulation also interferes with insulin secretion, although more studies are needed to elucidate the mechanism of action in humans [190].

Beyond fat ectopic depots, numerous studies have confirmed that also the increased accumulation of ECM components and the activation of several ECM receptor pathways in WAT are associated with IR and obesity-associated inflammation.

Relating to collagen, excessive accumulation can promote IR in humans [191]. Collagen is less soluble and less digestible by collagenases and cyanogen bromide in patients with diabetes compared to controls, which can increase the accumulation in different tissues, highlighting liver, bone and skeletal muscle [192,193,194]. The collagen depots produce the thickening of capillary basement membrane, a signal of diabetic microangiopathy, what precedes the T2D [195,196]. In fact, patients with diabetes are more probably to suffer a bulk of tendon diseases, such as tendinopathy than healthy individuals [197]. Several studies have demonstrated the association between IR and collagen accumulation. A recent publication has demonstrated a positive association between collagen content in WAT and the degree of IR in both Chinese and Caucasian populations [198]. Similar results were observed among obese subjects where insulin sensitivity was evaluated trough hyperinsulinemic-euglycemic clamp. The grade of fibrosis in WAT was higher in the most insulin-resistant subjects, which made the authors conclude that WAT fibrosis is associated with IR [199]. Another study also reinforced the idea that IR was followed by a high rise of type I and type III collagens in WAT biopsies of healthy males [199,200]. It has also observed that excessive collagen accumulation in WAT may inhibit angiogenesis [201]. These studies in humans propose a pathogenic role of collagen accumulation in insulin sensitivity, confirming the results obtained in various animal models of metabolic diseases [37,39].

Although the mechanisms through ECM remodeling are associated with IR is not completely known, some authors have proposed several mechanisms [202,203]. De Lin et al. [13] suggested a link between ECM receptor in WAT to obesity-associated IR. This pathway activation could induce genes expression implied in metabolically unfavorable processes, such as adipocyte death, angiogenesis inhibition and proinflammatory macrophage infiltration, which could result in IR.

First, it is thought that excessive accumulation of ECM components in WAT reduces the expansion of adipocytes and causes cell death through either necrosis or apoptosis [161]. Consequently, adipose inflammation and IR is caused due to the capacity of necrotic adipocytes to attract proinflammatory macrophages [204]. Second, as it has already reviewed herein, although an increase in angiogenesis is a necessary process for WAT expansion, this process is dysregulated in obesity. Lastly, immune cell infiltration into WAT provides an important link among obesity, IR and diabetes. WAT in insulin-resistant obese patients shows a major infiltration of macrophages compared with their respective controls, independently of the fat mass [205]. It has also been suggested that WAT inflammation may be a cause rather than the consequence of IR since progressive macrophage infiltration in VAT preceded an increase in insulin serum [205].

In conclusion, angiogenesis and ECM remodeling play crucial roles in WAT inflammation and novel therapeutic approaches are needed for effective treatment of IR and metabolic associated diseases such as T2D and CVD. Table 1 summarizes the studies related to extracellular matrix remodeling of adipose tissue in obesity and insulin resistance.

### 3.3. Potential Targets to Improve Adipose Fibrosis and Dysfunction in Obesity

As described above, obesity-induced adipose tissue expansion drives the continuous production and deposition of ECM, which is stated as ECM remodeling. The main consequence of this is fibrosis that impairs adipose tissue plasticity. However, this process is still investigating and there is not enough evidence to provide a potential therapeutic approach to preserve a healthy ECM under adipose tissue expansion. Some studies has been focused in antidiabetic drugs such as metformin or dipeptidyl peptidase-4 (DDP4) to treat T2D. It seems that metformin inhibits excessive ECM deposition in WAT of obese mice, decreasing the collagen deposition surrounding adipocytes and this is through AMP-activated protein kinase (AMPK) activation. AMPK is a kinase which is considered the master regulator of metabolism, activated by low cellular energy status and it may be a therapeutic target for the treatment of several metabolic diseases such as obesity, diabetes and cancer. Therefore, integrating AMPK activation may provide a potential therapeutic target to prevent collagen deposition, fibrosis in adipose tissue and whole-body IR in obesity [206]. In this line, it has been also demonstrated that metformin prevents hypoxia and reduces HIF1-α accumulation in adipose tissue. Hypoxia is a consequence of the enlargement of adipocytes and this limits oxygen from the vessels and adipocytes response by increasing HIF1-α, which is an indicator of adipose dysfunction [207].

DDP4 inhibitors target the enzymatic degradation of incretin peptides and it have been also recognized for its role to treat T2D. DDP4 has nonenzymatic functions that include its interaction with adenosine deaminase and other ECM proteins. Then, in addition to its well-known function in regulation of glucose homeostasis through its enzymatic functions, *DPP4* expression in inflammatory cells such as macrophages and dendritic cells might play a key role in regulating the inflammation in adipose tissue through the nonenzymatic function. Actually, *DPP4* expression is upregulated in adipose mononuclear immune cells in obesity-induced IR, which may help to a rapid DPP4-mediated degradation of incretin peptides via its enzymatic function. Understanding those functions of DPP4 might be a therapeutic target to treat T2D and also to prevent the inflammation, and in the end fibrosis, in adipose tissue [208].

In conclusion, those drugs are promising targets to treat metabolic diseases and future studies will explore whether metformin or DDP4 inhibitors could be used to improve the health of individuals with obesity or to prevent fibrosis and IR in adipose tissue.

## 4. Epigenetic

Epigenetic mechanisms control gene activity and the development of an organism [209,210]. The epigenome involves methylation of DNA, histone modifications, and RNA-mediated processes that regulate cell differentiation, gene expression, parental imprinting, X-chromosome inactivation, and the stability and structure of DNA. The disruption of this balance may cause several pathologies and contribute to obesity and T2D [211].

As we described above, WAT hypertrophy is associated with ECM function and angiogenesis. The formation of new adipocytes is constant during adulthood and this gives a support that epigenetic mechanisms could participate in regulation of adipose morphology associated to ECM remodeling. Indeed, a recent study in a large cohort of women has shown that CpG-methylation was related to adipose morphology on abdominal subcutaneous adipocytes. A higher proportion of CpG-sites were methylated in hypertrophic compared to hyperplastic WAT, where 2508 differentially methylated GpG-sites in 638 adipose morphology-associated genes. Interestingly, those genes were up regulated related to WAT hypertrophy, such as IR, lipolysis, ECM, and innate immunity. Therefore, methylation of CpG may be critical in determining adipose morphology and constitute a new target to treat T2D [212].

Another study has shown a global human methylome analyzed in visceral adipose tissue from morbidly obese patients, and they found that the genes associated with the largest methylation fold change were genes related to metabolic processes, proliferation, inflammation, and ECM remodeling [213]. On the other hand, subcutaneous WAT also has revealed genes and pathways differentially methylated within in monozygotic (MZ) twin pairs who are discordant for BMI. It has been revealed that DNA methylation of 17 genes and 26 pathways in subcutaneous adipose tissue were related to increased adiposity, demonstrating the combination of different pathogenic changes that characterize subcutaneous adipose tissue in obesity such as increased ECM remodeling, lipogenesis and inflammation. Therefore, it seems that subcutaneous adipose tissue needs to adapt to expand under higher energy intake in obesity and this is epigenetically regulate [214]. Regarding adipose tissue expansion, it has been also reported three CpG sites located within the hypoxia-inducible factor 3 subunit alpha (*HIF3A*), which is part of a group of heterodimeric transcription factors regulating responses to hypoxia [215]. Even though further studies are needed to understand the human epigenome, epigenetic modifications clamps promise for therapeutic strategies in obesity and metabolic diseases.

## 5. Clinical Studies

As we mentioned above, adipose tissue is the momentary storage of energy as triglycerides. When the energy balance is a positive energy balance, preadipocytes develop a huge cell shape change and they differentiate into adipocytes. In this process, ECM remodeling is crucial to allow a proper adipose tissue expansion [33]. In people with obesity, large-scale transcriptomic analyses of WAT revealed many inflammatory changes and genes that are significantly involved in several biological processes, either in stable weight conditions or during weight loss [216]. Khan et al. investigated the metabolic dysregulation of the body and found that T2D is related to changes in the ECM of adipose tissue [161]. Another study has shown that single-nucleotide polymorphisms in the promoter region of the *MMP-1* gene among Korean subjects are associated with BMI [217].

Transcriptomic analysis of the subcutaneous WAT from obese human subjects, in stable weight conditions and after weight loss following bariatric surgery has shown that ECM constituents were significantly upregulated, and also suggested that those elements could play a major role in local inflammatory phenomena to the alteration of WAT metabolic functions in those obese subjects [218]. In the case of the children population, children with overweight had significantly less total collagen compared to normal-weight children, whereas, collagen areas were not positive for COL6 and showed little evidence of collagen surrounding adipocytes [219].

Forty-six subjects with impaired fasting glycemia or impaired glucose tolerance and features of metabolic syndrome were recruited for a randomized controlled and individualized weight reduction intervention. In the weight reduction group downregulation of gene expression involving ECM and cell death was seen. Such changes did not occur in the control group [220]. Healthy participants with overweight or obesity followed either a 5-week very low calorie diet or a 12-week low-calorie diet with a subsequent 4-week weight stabilization period and a 9-month follow-up. Changes in leukocyte integrin gene activity and ECM remodeling were observed [221]. A follow up study during 5 weeks with very-low-calorie diet and subsequent 4-week weight-stable diet showed a correlation between stress and ECM-related genes, being highly related to weight regain in adipose tissue biopsies [222].

In addition, the genetic variation in ECM-related genes was investigated in participants with overweight and obesity of the European DiOGenes study that received an 8-week low-calorie diet with a 6-month follow-up. The risk of weight regain was increased by the gene variation in *POSTN, LAMB1, COL23A1 FBLN5,* and *FN1* genes [223].

Forty-four healthy men were involved in an overfeeding protocol with a lipid-enriched diet for 2 months. Subcutaneous abdominal adipose tissue was in the basal state after 14 days and at the end of the protocol. More than 60 genes encoding proteins of ECM were upregulated such as collagens, adhesion proteins, proteoglycans, and MMP-2, -9, and -15. This intensive regulation suggests that ECM remodeling is highly involved during weight gain [224] and implies COL6A3 in adipose tissue expansion [36,161]. Other clinical trial was carried out in forty healthy individuals overfed for 28 days and skeletal muscle biopsies were taken at baseline, day 3, and day 28. Muscle COL1 and COL3 and *MMP-2* mRNA levels were significantly higher 28 days after overfeeding, with no significant changes in *MMP-9, TIMP-1, CD68,* and integrin expression. Microarray-based gene set tests shown that pathways related to ECM receptor interaction, focal adhesion were significantly altered [225]. Table 2 summarizes the clinical studies related to extracellular matrix remodeling.

## 6. Concluding Remarks and Future Perspectives

As we have discussed above, ECM remodeling is a requirement for healthy adipose tissue expansion. This process also includes the formation of new blood vessels to prevent fibrosis, inflammation and, ultimately, adipose tissue dysfunction and IR. Thus, an inappropriate ECM remodeling happens in both humans and rodents with obesity, IR and T2D. However, the precise mechanism involved in this process is still unknown; even though some hypothesis has been proposed. On the other hand, integrin signaling are the main tissue receptors that transduce the signaling from the outside into the cells and are critical in adipose tissue expansion. Further studies are needed to determine the mechanisms underlying diet-induced ECM and insulin signaling in adipose tissue, in which epigenetic modifications could be a novel strategy in the treatment of obesity and metabolic diseases in new and innovative clinical trials.

## Figures and Tables

**Table 1:** Main characteristics of studies related with angiogenesis.

Measure/. Reference	Effect in Blood Levels/WAT	Fluid or Tissue	Sample	Other Effects
VEGF [131]	Elevated in patients with obesity	Serum	10 men and 28 women, all of them with obesity	VEGF-A serum was reduced after weight reduction. VEGF-A was positively associated with visceral fat accumulation and BMI
VEGF [133]	Elevated in patients with obesity	Plasma	15 obese and 15 normal-weight men	VEGF-A positively associated with BMI
VEGF [134]	No change	Serum	21 (13 women/ 8 men) lean and 44 (32 women/ 12 men) obese	
VEGF-A [136]	Decreased in obesity	WAT	Obese mice	
VEGF [137]	Decreased in patients with obesity	WAT	9 (5 men/4 women) lean and 12 (6/6) obese	*VEGF-A* expression negatively associated with capillary density
VEGF-A [138]	-	-	C57Bl6/SJL mice	*VEGF-A* overexpression protected mice from HFD inflammation and IR
VEGF-A [56]	Overexpression in patients with obesity	WAT	26 obese and 17 normal-weight men	*VEGF-A* expression was higher in low IR obese than in high IR patients
PO_2_ [150]	Decreased in obesity	WAT	23 obese and 21 lean men	
PO_2_ [151]	Decreased in obesity	WAT	24 (20 women/4 men) obese and 10 lean (7 women/3 men)	
PO_2_ [153]	No differences	WAT	7 lean (5 women/2 men), 7 obese women	
PO_2_ [137]	Decreased in obesity	WAT	9 lean (4 women/5 men), 12 (6/6) overweight and obese	
PO_2_ [152]	No differences	WAT	7 lean men, 28 (14 women/14 men) obese	Abdominal subcutaneous AT oxygenation is associated with insulin sensitivity
PO_2_ [147]	Elevated in obesity	WAT	10 lean, 10 obese men	

Abbreviations: AT: adipose tissue; BMI: body mass index; HDF: high-fat diet; IR: insulin resistance; PO_2_: partial pressure of oxygen; VEGF: vascular endothelial growth factor; WAT: white adipose tissue.

**Table 2:** Main characteristics of clinical studies.

Reference	Population	Sample	Main Results
Nho et al. [217]	Population-based cohort study consisting of 530 subjects	One group with BMI <25.0 and the other BMI ≥25.0, and MMP-1 polymorphisms by pyrosequencing analysis were measured.	MMP-1 frequencies were significantly higher in subjects with BMI <25.0
Henegar et al. [218]	Fifty five obese subjects and 15 lean controls were prospectively recruited	Transcriptomic signature of the subcutaneous WAT in obese human subjects was analyzed	Phenotypic alterations of human pre-adipocytes may lead to an excessive synthesis of ECM components
Tam et al. [219]	65 otherwise healthy children having elective surgery were selected	Collagen (total and pericellular), and ECM gene expression markers were measured	Increased collagen in AT is associated with BMI z-score, suggesting dynamic interaction between ECM remodeling and immune cells even at an early age.
Kolehmainen et al. [220]	Forty-six subjects with metabolic syndrome were randomized either to a weight reduction (*n*=28) or a control (*n*=18) group lasting for 33 weeks.	Subcutaneous AT biopsies were performed using microarray technology	Genes regulating the ECM and cell death showed a strong downregulation after long-term weight reduction
Roumans et al. [221]	61 healthy overweight or obese participants followed either a very-low-calorie diet or a low-calorie diet	Abdominal subcutaneous AT biopsy samples were collected for microarray analysis	ECM modification seems to be involved
Roumans et al. [222]	31 participants with overweight or obesity followed a 5-week very-low-calorie diet with a subsequent 4-week weight-stable diet, and then an uncontrolled 9-month follow-up.	AT biopsies were collected for microarray analysis.	Interaction analysis between stress- and ECM-related genes revealed that several gene combinations were highly related to weight regain.
Roumans et al. [223]	469 overweight and obese subjects were on an 8-week low-calorie diet with a 6-month follow-up.	AT biopsies were collected for microarray analysis.	Variants of ECM genes are associated with weight regain after weight loss in a sex-specific manner.
Alligier et al. [224]	Forty-four healthy men were involved in an overfeeding protocol with a lipid-enriched diet for 2 months.	Subcutaneous abdominal AT biopsies were taken	Reorganization of gene expression patterns occurred in AT with an upregulation of numerous genes involved in angiogenesis and ECM remodeling.
Tam et al. [225]	Forty healthy individuals were overfed by 1,250 kcal/day for 28days.	Skeletal muscle biopsies were taken	Skeletal muscle ECM remodeling occurs early in response to over-nutrition with as little as 3% body weight gain.

Abbreviations: AT: adipose tissue; BMI: body mass index; ECM: extracellular matrix; MMP: matrix metalloproteases; WAT: white adipose tissue.
